# Growth Phase-Dependent Changes in the Carbohydrate Metabolism of Penicillium Strains from Diverse Temperature Classes in Response to Cold Stress

**DOI:** 10.3390/ijms26199308

**Published:** 2025-09-24

**Authors:** Jeny Miteva-Staleva, Ekaterina Krumova, Maria Angelova

**Affiliations:** Department of Mycology, The Stephan Angeloff Institute of Microbiology, Bulgarian Academy of Sciences, Acad. G. Bonchev Str. bl.26, 1113 Sofia, Bulgaria; j_m@microbio.bas.bg

**Keywords:** oxidative stress, filamentous fungi, Antarctica, carbohydrate metabolism, hexokinase, glucose-6-phosphate-dehydrogenase, glyceraldehyde-3-phosphate dehydrogenase, isocitrate dehydrogenase, succinate dehydrogenase, malate dehydrogenase

## Abstract

Three fungal strains belonging to the genus Penicillium from different temperature classes (two Antarctic strains—psychrotolerant and mesophilic, and a temperate mesophilic) were used to investigate their metabolic cell response to cold stress. The exponential- and stationary-growth-phase fungal cultures were exposed to a transient temperature downshift from optimal to 6 and 15 °C, respectively. The activity of the enzymes hexokinase, glucose-6-phosphate dehydrogenase, and glyceraldehyde 3-phosphate dehydrogenase from the glycolytic pathway, and that of the enzymes isocitrate dehydrogenase, succinate dehydrogenase, and malate dehydrogenase from the TCA cycle were studied. In all experiments, the cold-induced oxidative stress increased the indicated enzymatic activities depending on the strain’s temperature characteristics, the degree of stress, and the growth phase. Furthermore, enzyme activity was lower in cells from stationary-phase cultures (older cells) compared to those from exponential-phase cultures (younger cells). The cellular response was more pronounced in mesophilic strains, regardless of the location of isolation. The cold-adapted Antarctic psychrotolerant strain exhibited enhanced tolerance to low-temperature stress compared to mesophilic strains. These findings emphasize the significance of temperature preferences and growth phase in the survival of fungi under conditions of cold-induced oxidative stress. New information could prove beneficial in forecasting the behaviour of fungal pathogens such as plant pathogens in agriculture and human pathogens in medicine.

## 1. Introduction

Ageing is an ongoing biological process that is reflected in morphological, physiological, and biochemical changes [1]. Despite ongoing debates, it is widely accepted that ageing results from the accumulation of irreparable damage at morphological, physiological, and molecular levels, ultimately leading to functional decline. This process was first described in eukaryotes and has since been reported in some bacteria and fungi [2]. Various species of filamentous fungi exhibit signs of ageing, including cellular senescence and a limited lifespan due to programmed degeneration [3].

There exists a multitude of theories aimed at explaining this phenomenon. The most notable and widely debated is the free radical theory of ageing introduced by Harman in 1956 [4,5]. According to this theory, the detrimental effects of reactive oxygen species (ROS) induce changes in cellular function throughout their lifespan. Later, this theory was modified into the oxidative stress theory of ageing to include other oxidants, such as peroxides and aldehydes, which are not radicals [6]. The relationship between ROS and ageing has been confirmed in a variety of organisms, including plants [7], animals [8], yeast [9], fungi [10], bacteria [11] and others. In general, the published data demonstrated that this relationship is very complex and depends on the experimental conditions.

The basis of the oxidative stress theory of ageing is the generation of ROS in aerobic cells. These ROS are molecules that can form due to the incomplete reduction in molecular oxygen during respiration. They included superoxide radicals (^●^O_2_^−^), hydrogen peroxide (H_2_O_2_), hydroxyl radicals (OH•), singlet oxygen (^1^O_2_), peroxyl radical (LOO^●^), alkoxyl radical (LO^●^), lipid hydroperoxide (LOOH), peroxynitrite (ONOO−), hypochlorous acid (HOCl), and ozone (O_3_), among others [12]. Depending on the presence or absence of unpaired electrons, they are classified as free radicals (^●^O_2_^−^, OH•, LOO^●^, and LO^●^) and non-free radicals (H_2_O_2_, HOCl, and O_3_), respectively [13]. Under normoxic conditions, ROS may play an important role in intracellular signalling, affecting growth, development, and metabolome regulation [14]. In the cases of increased ROS levels, an immediate neutralization is required by the antioxidant enzyme systems [12].

However, when the concentration of ROS exceeds the physiological capacity of the cell, an imbalance between pro- and antioxidants, with a predominance of the former, results in oxidative stress (OS) [15,16]. These ROS, which avoid antioxidant defence, have the potential to cause severe injury to all cellular elements, including lipids, proteins, and nucleic acids. Furthermore, they have been identified as playing a pivotal role in the development of numerous age-related and degenerative diseases, including Alzheimer’s disease, Parkinson’s disease, atherosclerosis, diabetes, and cell atrophy, among others [17,18]. The generation of ROS, which contributes to oxidative stress, is accelerated by exposure to multiple environmental factors, including UV rays, heavy metals, pesticides, xenobiotics, temperature changes, osmotic shock, and so forth. Sources of the so-called “radical pollution” are also neutrophils, macrophages, dendritic cells, and monocytes, which use ROS against various pathogens during microbial invasion [19].

Among all environmental factors, extreme temperatures hold the utmost significance for the realization of OS. Temperature stress results in an elevation of intracellular ^●^O_2_^−^ and H_2_O_2_ levels [20]. Low temperatures impede the rate of enzymatic and transport processes within cells, leading to a reduction in adenosine triphosphate (ATP) consumption and the accumulation of electrons at certain stages of the respiratory chain [21]. Consequently, this triggers a substantial rise in ROS levels [22] and disrupts the antioxidant balance of the organism, resulting in OS [23].

The relationship between low temperature and OS has been evidenced by a number of experiments. Most findings regarding enhanced ROS levels and subsequent morphological, physiological, and metabolic events after exposure to cold temperatures have been published for plants [16,24,25] and human cells [26,27]. Similar results have been demonstrated in archaea [21] and bacteria [28], but fungi have not been well investigated. Additionally, the cold stress response exhibited by fungi isolated from habitats characterized by extreme cold is more rarer [23]. The temperature reduction from 30 to 10 °C induced ROS generation and activated antioxidant defence in *Saccharomyces cerevisiae* [29]. The same response has been established for *Aspergillus flavus* [30], *Pseudogymnoascus pannorum* [31], oleaginous yeast *Rhodosporidium kratochvilovae* [32], etc.

It is well known that exposure to low temperatures can induce conformational changes in protein structure, which have been demonstrated to perturb the conventional progression of enzymatic reactions, membrane functions (transport, cell division), and the processes of transcription, translation, replication, and mRNA folding [33]. As posited by Riccardi et al. [34], exposure to low temperatures induce alterations in the activity of pivotal metabolic enzymes implicated in glycolysis, the tricarboxylic acid (TCA) cycle, and the biosynthesis of amino acids, nucleotides, and cofactors. Recent studies have revealed that carbon metabolism is redirected towards the pentose-phosphate metabolic pathway (PPP) [35,36]. Concomitantly, glycolysis and downstream pathways, such as the tricarboxylic acid (TCA) cycle, are suppressed. The stimulation of PPP has been demonstrated to increase nicotinamide adenine dinucleotide phosphate (reduced form) (NADPH) production, thereby maintaining antioxidant enzyme activity. Lehmann et al. [37] proposed that a combination of transcriptional changes (induction of antioxidant enzymes and defence mechanisms) and posttranslational control of carbon flux via central carbon metabolism is required to cope with oxidative stress conditions. It should be noted that there are very few published reports on TCA cycle enzyme activities in cold-adapted fungi. The accumulation of TCA cycle metabolites has been evaluated in the Antarctic yeast *Mrakia blollopis* [38,39], the cryptoendolithic black fungus *Cryomyces antarcticus* [40], and *Flammulina velutipes* [41]. However, comparative studies on the role of the TCA cycle in the cellular response to cold-induced stress in fungi from polar and temperate habitats are lacking.

The existing data regarding the relationship between cold stress, metabolic response, and the ageing process is both limited and ambiguous. Moreover, there is a lack of information on organisms isolated from extremely cold habitats, as well as comparative data on mesophilic temperate microorganisms and analogous ones isolated from polar regions.

Fungi are a very beneficial object to study age-related changes after exposure to extreme factors that induce ROS generation. These organisms grow rapidly, form abundant mycelium, demonstrate short generation times, and synthesize a wide range of enzymes. In addition, fungi exhibit a pronounced cellular response to stress, and ageing processes are visible at the microscopic and macroscopic levels. Furthermore, fungi are classified as eukaryotic organisms, and the findings derived from the study can be extrapolated to higher eukaryotes.

In our previous studies, we evaluated the cold cell response of three fungal strains (two strains isolated from Antarctica and one from a temperate region) belonging to the genus Penicillium. The results demonstrated that the drastic reduction in growth temperature below the optimal caused a significant induction in ROS levels and oxidative stress events during exponential and stationary growth phases in a temperature- and growth-phase-dependent manner [42,43,44].

The present study is a continuation of the above-mentioned research. It was focused on the cell response of the same three penicillium strains against cold stress on the metabolite level. We hypothesize that (1) the cell response to low temperatures evolves changes in activity of basic metabolite pathways, supplying the culture with energy and metabolites; (2) the cold-adapted fungi are more tolerant to stress than their mesophilic counterpart; (3) metabolic changes are characteristic of both growth phases. The aim was to assess the effect of transient temperature downshift on the enzyme activity of main metabolite pathways during the exponential and stationary growth phases of both Antarctic strains—psychrotolerant *Penicillium olsonii* p14 and mesophilic *Penicillium waksmanii* m12, as well as the temperate mesophilic strain *Penicillium rugulosum* t35. To the best of our knowledge, this is the first study to investigate the involvement of carbohydrate metabolism and tricarboxylic acid cycle enzymes in the response to cold stress in cells from exponential-phase (younger) and stationary-phase (older) cultures isolated from Antarctica.

## 2. Results

**Figure 1:**
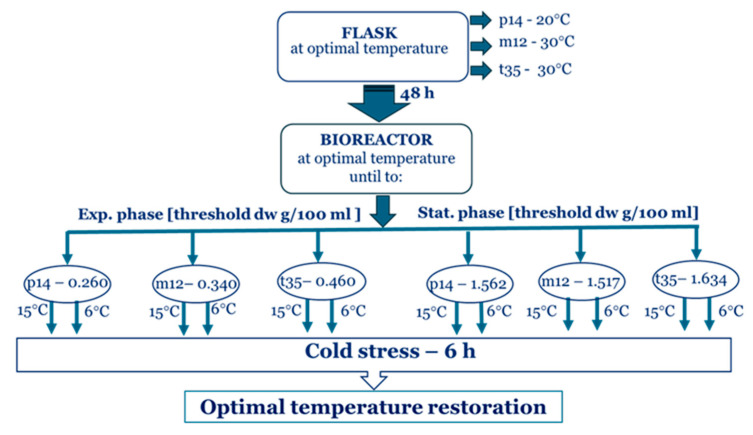
Schematic diagram of the experimental design.

The model penicillium strains were cultivated at their respective optimal temperatures until they reached the middle exponential or middle stationary growth phases. The cultures were then subjected to a temperature reduction of 15 °C or 6 °C for 6 h, followed by restoration of the optimal temperature for a further 4 h. The experimental design is presented in Figure 1.

### 2.1. Effect of Cold Stress on the Activity of Glycolytic Enzymes

Different types of stress trigger biochemical and molecular responses, including the up- or downregulation of enzymes in the main metabolic pathways, in order to counteract the adverse effects [45]. Among the enzymes of glycolysis, changes in the activity of hexokinase (HK), glucose-6-phosphate-dehydrogenase (G6PDH), and glyceraldehyde-3-phosphate dehydrogenase (GAPDH) in the exponential and stationary phases of the three model fungal strains were studied.

#### 2.1.1. Changes in HK Activity

**Figure 2:**
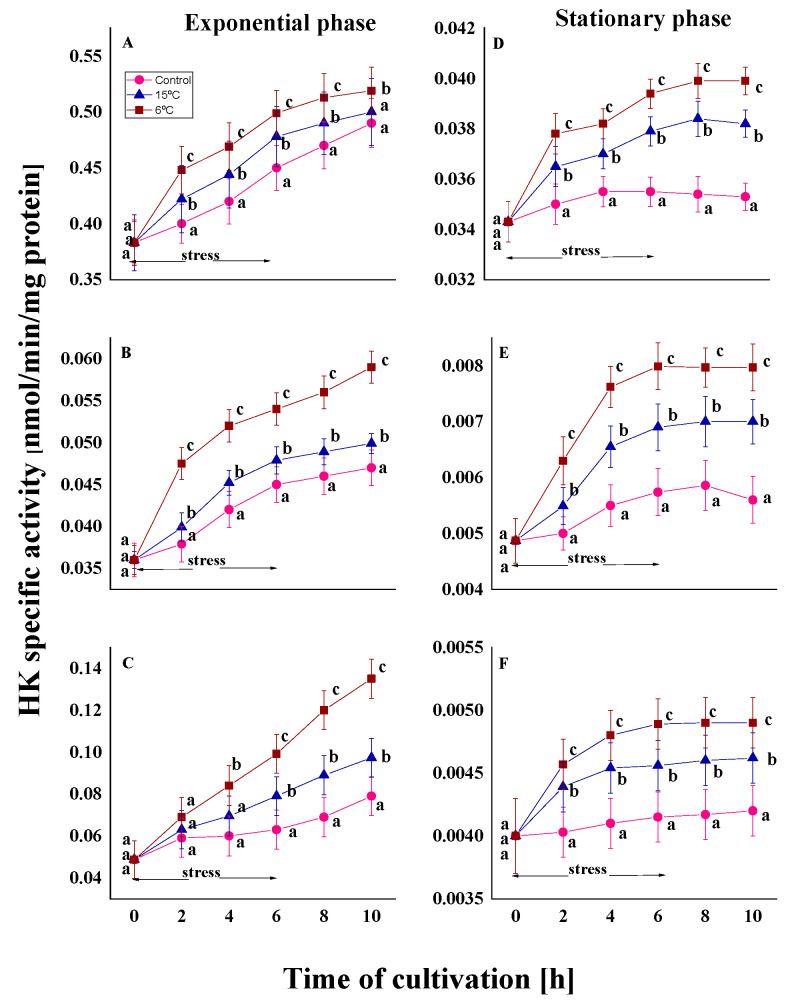
HK activity in the psychrotolerant strain *P. olsonii* p14 (**A**,**D**), the Antarctic mesophilic strain *P. waksmanii* (**B**,**E**), and the temperate *P. rogulosum* t35 (**C**,**F**) during the exponential (**A**–**C**) and the stationary (**D**–**F**) growth phase. Enzyme activity was determined at the corresponding optimal temperature (●) and at a decrease in temperature from optimal to 6 °C (■) or to 15 °C (▲). The values are the means of three repeated experiments, each consisting of three parallel runs. The bars represent the standard deviation. The temperature downshift turns out to have a statistically significant effect (*p* < 0.05). Different lower letters indicate significant differences (Tukey’s test *p* < 0.05) between cold stress treatments and the control variants at the same period of cultivation.

The enzyme HK is responsible for catalyzing the first step of glycolysis by phosphorylating glucose to generate glucose-6-phosphate [46]. The untreated variants of the three strains during the exponential growth phase reveal a rapid enhancement in enzyme activity over the cultivation period. At the end of the experiment (10th hour), HK activity was found to be 1.3 to 1.6 times higher than the initial measurement (Figure 2A–C).

As expected, the stationary phase control cultures exhibited significantly lower values of the HK activity compared to the exponential phase. This trend was observed both at the beginning of the experiment and throughout the cultivation process. The enzyme level in old cells under optimal cultivation conditions was found to be 12- to 17-fold lower than the corresponding variants in young cells at the same exposure time (Figure 2D–F).

In all experiments with the induction of oxidative stress as a result of a sharp decrease in temperature (from optimal to 15 or 6 °C), an increase in HK activity was observed depending on the temperature characteristics of the strains, the degree of stress, and the growth phase of the culture. A more significant increase in enzyme activity was observed in the mesophilic strains, *P. waksmanii* m12 and *P. rugulosum* t35, under stress conditions compared to the psychrotolerant strain, *P. olsonii* p14. This increase was observed in both phases of development. The Antarctic mesophilic strain exhibited the highest activity during the experiment relative to the initial level, ranging from 2 to 2.7 times in the exponential phase and 1.5 to 1.6 times in the stationary phase. The increase in the other mesophilic strain was, respectively, 1.4-, 1.6-, and 1.2-fold for both phases of development. These differences in cellular response also demonstrate dependence on the habitat of isolation.

The results also indicated that the degree of stress is key to changes in enzyme activity. During the 6 h stress period, the specific activity of HK increased sharply in the first 2 h. Variants in which the temperature was reduced from the optimal level to 6 °C showed a significantly sharper increase throughout the cultivation process than those at 15 °C. While maximum HK activity in cells treated at 15 °C is approximately 1.2 times higher than in the control variants, reducing the temperature to 6 °C leads to a more pronounced increase (1.3- and 1.7-fold). The noted difference in the activity was more pronounced after 6 h of stress exposure. Both growth phases showed a rise in HK activity, confirming the temperature-dependent effect of cold exposure, based on the calculated percentage increase in treated cultures compared to controls after 6 h of stress (Supplementary Figure S1).

The present study also considers the role of the growth phase. Under conditions of low-temperature stress, the activity of HK increased in both young and old cells of the three strains studied. In the stationary phase, the level of the enzyme was significantly lower than in the exponential phase (see Figure 2). However, stress caused an increase in activity in both phases, although this was less pronounced in old cells. As can be seen in Figure 2 (D–F, stationary phase), after the second hour, the level of enzyme activity remains unchanged and plateaus before the stress effect ceases. In cells from the exponential phase, this increase continued even after the stress effect had ceased (from the 6th to the 10th hour) (Figure 2A–C)

#### 2.1.2. Changes in G6PDH Activity

**Figure 3:**
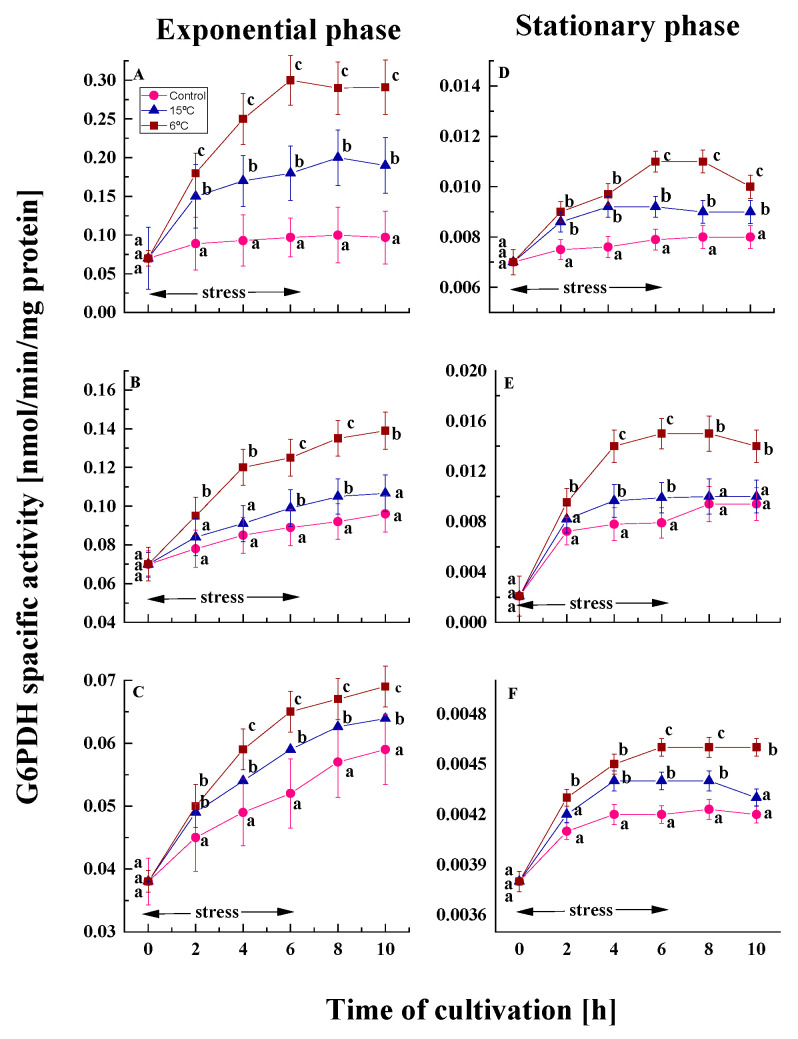
G6PDH activity in the Antarctic psychrotolerant strain *P. olsonii* p14 (**A**,**D**), the Antarctic mesophilic strain *P. waksmanii* m12 (**B**,**E**) and the temperate *P. rogulosum* t35 (**C**,**F**) during the exponential (**A**–**C**) and the stationary (**D**–**F**) growth phase at optimal temperature (●) and upon temperature decrease from optimal to 6 °C (■) or to 15 °C (▲). The values are the means of three repeated experiments, each consisting of three parallel runs. The bars represent the standard deviation. The temperature downshift turns out to have a statistically significant effect (*p* < 0.05). Different lower letters indicate significant differences (Tukey’s test *p* < 0.05) between cold stress treatments and the control variants at the same period of cultivation.

G6PDH catalyzes the initial step in the pentose phosphate pathway (PPP), which is a source of significant metabolites, including an additional reserve of reducing power in the form of NADPH [46]. The trend of reduced enzyme activity in stationary phase cells compared to exponential phase controls was also observed for this enzyme (Figure 3). For all variants of the studied strains, enzyme activity was 10- to 14-fold lower in old cultures than in young cultures at the end of the cultivation period.

Under conditions of low-temperature stress, cells from the exponential phase of the three model strains demonstrated an increase in the activity of the G6PDH enzyme. A temperature downshift to 15 or 6 °C for six hours resulted in an augmentation of enzyme activity in young cells—1.9- and 3.0-fold in *P. olsonii* p14, 1.1- and 1.4-fold in *P. waksmanii* m12, and 1.1- and 1.2-fold in *P. rogulosum* t35 in comparison to the control (Figure 3A–C). The most substantial increase was observed in the psychrotolerant strain, while the two mesophilic strains did not demonstrate any significant differences between them. In this case, the activity of G6PDH does not depend on the isolation habitat, but rather on the degree of stress and the temperature characteristics of the strain.

Treatment of stationary phase cells at low temperature increased G6PDH activity (Figure 3D–F). However, the level of the enzyme was significantly lower than in the exponential phase. In old cultures of the model strains, a clear positive correlation between enzyme activity and the degree of stress was also evident.

Using the data from Figure 3, the relative G6PDH activity was calculated by comparing the treated and control cultures after six hours of exposure to a low temperatures (Supplementary Figure S2). The findings show that stress increases activity in both phases, depending on its intensity.

#### 2.1.3. Changes in Glyceraldehyde Phosphate Dehydrogenase Activity

**Figure 4:**
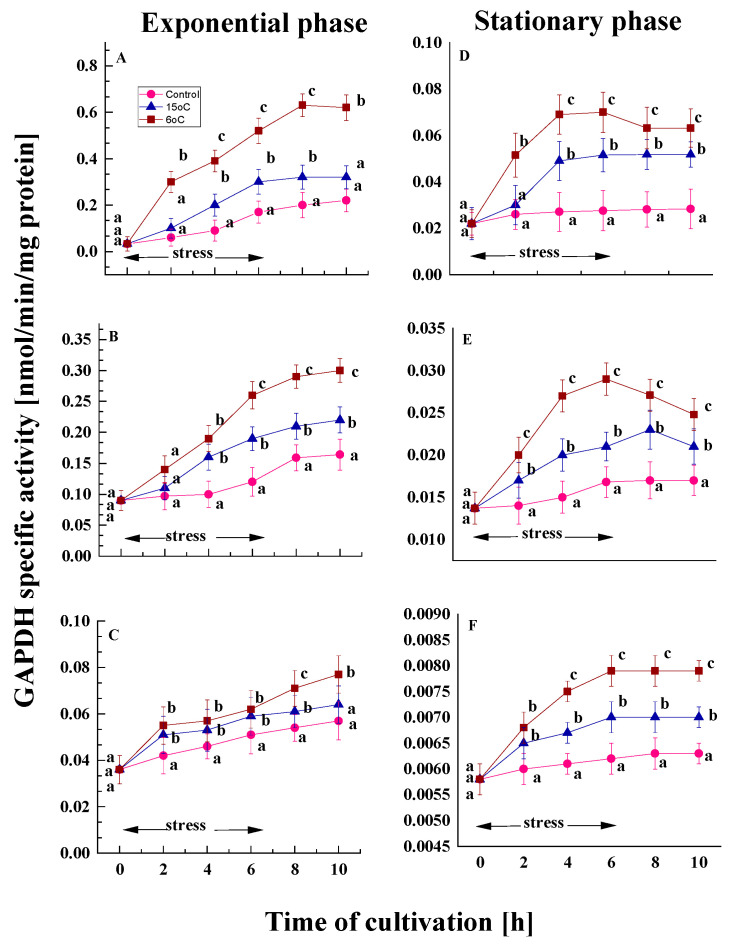
The changes in GAPDH activity in the Antarctic psychrotolerant strain *P. olsonii* p14 (**A**,**D**), the Antarctic mesophilic strain *P. waksmanii* m12 (**B**,**E**) and the temperate *P. rogulosum* t35 (**C**,**F**) during the exponential (**A**–**C**) and the stationary (**D**–**F**) growth phase at optimal temperature (●) and upon temperature decrease from optimal to 6 °C (■) or to 15 °C (▲). The values are the means of three repeated experiments, each consisting of three parallel runs. The bars represent the standard deviation. The temperature downshift turns out to have a statistically significant effect (*p* < 0.05). Different lower letters indicate significant differences (Tukey’s test *p* < 0.05) between cold stress treatments and the control variants at the same period of cultivation.

GAPDH is crucial for energy production within the cell. The enzyme catalyzes reversible oxidation and phosphorylation reactions in glycolysis [46]. A comparison of the activity of GAPDH in the control variants of the model strains from both growth phases (Figure 4) revealed some differences. Throughout the cultivation process, the level of the enzyme increased in young cells; however, cells from the stationary phase exhibited almost constant GAPDH activity until the end of the experiment. Furthermore, untreated young cultures maintained nearly stable GAPDH activity in comparison to untreated cultures from the stationary phase.

The cold stress caused an increase in the activity of the GAPDH enzyme in the three model strains in the exponential phase (Figure 4A–C). Young cells of the psychrotolerant strain *P. olsonii* p14 demonstrate 1.8- and 3.0-fold higher GAPDH activity at 15 and 6 °C, respectively, compared to the control variant at the end of the stress (Figure 4A). These conditions caused up to 1.6- and 2.1-fold increase in the activity of the enzyme in the Antarctic mesophilic strain (Figure 4B). The temperate mesophilic exhibited a smaller increase in activity (1.2- to 1.3-fold) compared to the control (Figure 4C). However, a more pronounced difference between the two stress temperatures appears later in time. A clear dependence of the enzyme activity on the degree of stress and on the thermal characteristics of the strain is outlined.

All variants of stationary-phase cultures demonstrated lower GAPDH levels compared to their corresponding exponential-phase variants (Figure 4). However, the impact of the transient temperature downshift to 15 and 6 °C on aged cells exhibited a comparable effect to that observed on young cells, albeit to a considerably lesser extent (Figure 4D–F).

The data confirmed that GAPDH activity was upregulated during the six-hour cold exposure period compared to the control variants. Enhanced relative activity was observed for all three fungal strains and both growth phases (Supplementary Figure S3).

### 2.2. Effect of Cold Stress on the Enzymes Activity of TCA Cycle

The changes in the activity of three enzymes from the TCA cycle—isocitrate dehydrogenase (ICDH), succinate dehydrogenase (SDH), and malate dehydrogenase (MDH) were studied under conditions of cold stress in two of the growth phases of the model strains. It was observed that, for all three enzymes evaluated, the levels of each enzyme were consistently lower in the control samples from the stationary phase than in the exponential phase throughout the entire growing period.

#### 2.2.1. Effect on the ICDH Activity

ICDH is a key source of NADP for the antioxidant system, which is essential for cell survival in the face of oxidative stress [47]. In the optimal conditions, all tested strains demonstrated lower ICDH activity during the exponential growth phase compared to the stationary one. This decline in activity was more pronounced among the mesophilic strains.

**Figure 5:**
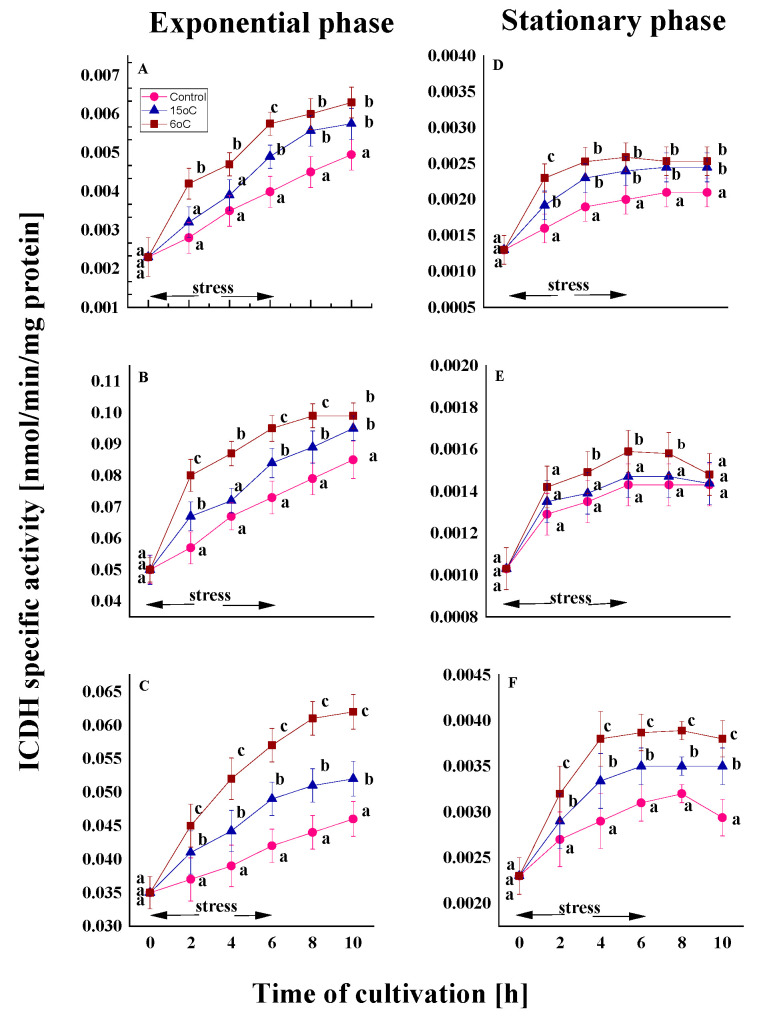
ICDH activity in the Antarctic psychrotolerant strain *P. olsonii* p14 (**A**,**D**), the Antarctic mesophilic strain *P. waksmanii* m12 (**B**,**E**) and the temperate *P. rogulosum* t35 (**C**,**F**) during the exponential (**A**–**C**) and the stationary (**D**–**F**) growth phase at optimal temperature (●) and upon temperature decrease from optimal to 6 °C (■) or to 15 °C (▲). The values are the means of three repeated experiments, each consisting of three parallel runs. The bars represent the standard deviation. The temperature downshift turns out to have a statistically significant effect (*p* < 0.05). Different lower letters indicate significant differences (Tukey’s test *p* < 0.05) between cold stress treatments and the control variants at the same period of cultivation.

Nonetheless, the temperature downshift induced alterations in both growth phases. In the exponential phase variants, an increase of approximately 1.2–1.4 times compared to the control was observed by the end of the experiment (Figure 5A–C). A comparable increase was also demonstrated by old cells, although to a significantly lesser extent (Figure 5D–F). In all cases, the cellular response was more pronounced at 6 °C than at 15 °C. The findings indicate that the reported change depends only on the degree of stress and is not influenced by the temperature characteristics of the strains. After restoration of optimal growth conditions, young cells continue to exhibit an increased tendency to activate the enzyme.

The assessed relative activity revealed an elevation in ICDH levels in cold-treated cultures compared to the control variants (see Supplementary Figure S4). A significant percentage rise was observed following treatment at both temperatures, with a more pronounced effect at 6 °C. Cold stress affected both young and old cells across all penicillium strains.

#### 2.2.2. Effect on the SDH Activity

The mitochondrial enzyme SDH catalyzes the oxidation of succinate to fumarate and provides electrons for the respiratory chain, due to its unique redox properties [47]. In combination with ubiquinone, it functions as a major antioxidant enzyme controlling the neutralization of superoxide radicals in mitochondria [48].

**Figure 6:**
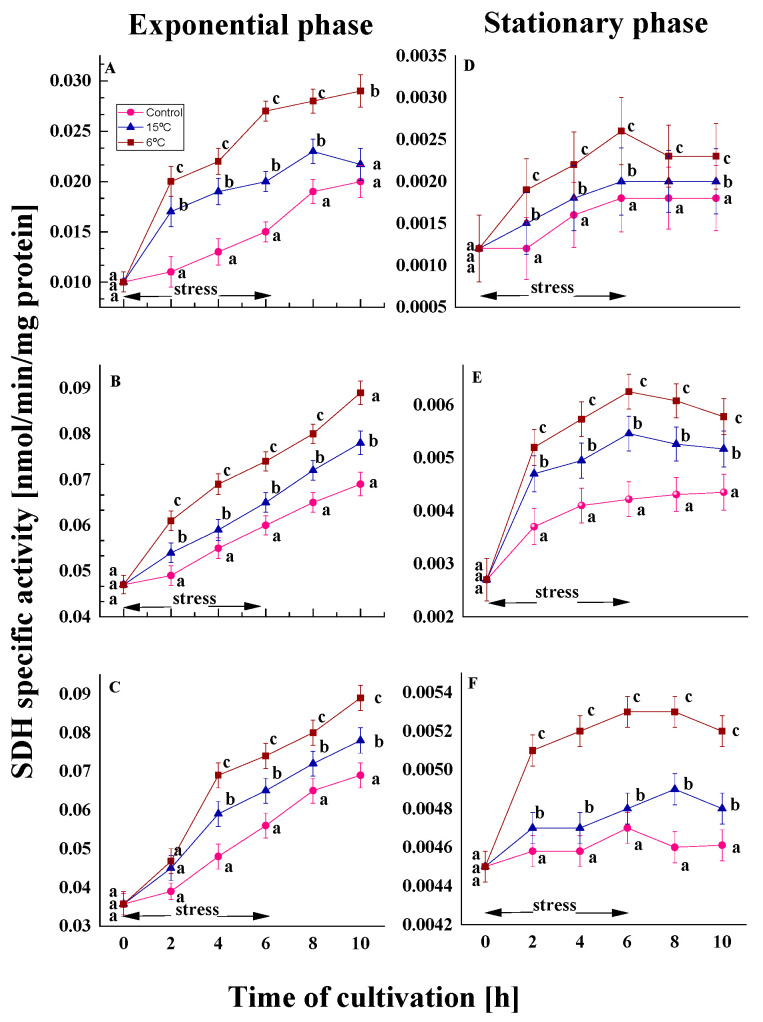
SDH activity in the Antarctic psychrotolerant strain *P. olsonii* p14 (**A**,**D**), the Antarctic mesophilic strain *P. waksmanii* m12 (**B**,**E**) and the temperate *P. rogulosum* t35 (**C**,**F**) during the exponential (**A**–**C**) and the stationary (**D**–**F**) growth phase at optimal temperature (●) and upon temperature decrease from optimal to 6 °C (■) or to 15 °C (▲). The values are the means of three repeated experiments, each consisting of three parallel runs. The bars represent the standard deviation. The temperature downshift turns out to have a statistically significant effect (*p* < 0.05). Different lower letters indicate significant differences (Tukey’s test *p* < 0.05) between cold stress treatments and the control variants at the same period of cultivation.

The temperature downshift induced alterations in both growth phases. In the exponential phase variants, an increase of approximately 1.2–1.3 times compared to the control was observed by the end of the experiment (Figure 6A–C). A comparable increase was also demonstrated by old cells, although to a significantly lesser extent (Figure 6D–F). In all cases, the cellular response was more pronounced at 6 °C than at 15 °C. The findings indicate that the reported change depends only on the degree of stress and is not influenced by the temperature characteristics of the strains. After restoration of optimal growth conditions, young cells continue to exhibit an increased tendency to activate the enzyme.

It is noteworthy that in untreated cultures, the activity of SDH in stationary phase cells is approximately 15 times lower than that observed in exponential phase cells. Following a period of six hours of cultivation, no significant changes were observed in comparison to the initial level of activity. However, after a sharp decrease in growth temperature, a trend towards an increase in enzyme activity was observed in cultures from both growth phases. A clear relationship between the enzyme activity and the degree of stress was evident. Cultures from the exponential phase of the psychrotolerant strain *P. olsonii* demonstrated 1.1- and 1.4-fold higher SDH activity at 15 and 6 °C, respectively, compared to the control (Figure 6A). Following the restoration of optimal growth conditions, a decline in enzyme activity was observed. In the variants exposed to 15 °C, the activity of the SDH was reduced to the level of the controls. In the mesophilic *P. waksmanii* and *P. rigulosum*, cold stress led to 1.1- and 1.3-fold higher SDH activity compared to the control (Figure 6B,C).

The above-noted findings were confirmed by data on relative SDH activity, which was measured in temperature-treated cultures versus the control group at the end of stress exposure (Supplementary Figure S5).

#### 2.2.3. Effect on the Malat Dehydrogenase Activity

**Figure 7:**
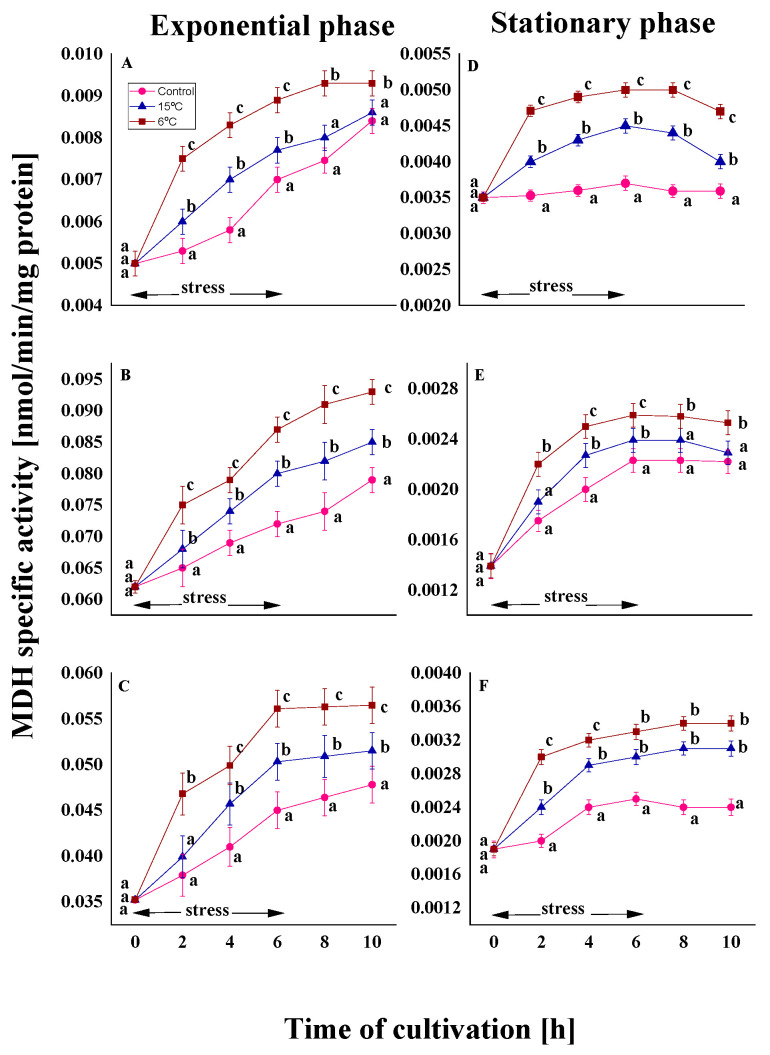
MDH activity in the Antarctic psychrotolerant strain *P. olsonii* p14 (**A**,**D**), the Antarctic mesophilic strain *P. waksmanii* m12 (**B**,**E**) and the temperate *P. rogulosum* t35 (**C**,**F**) during the exponential (**A**–**C**) and the stationary (**D**–**F**) growth phase at optimal temperature (●) and upon temperature decrease from optimal to 6 °C (■) or to 15 °C (▲). The values are the means of three repeated experiments, each consisting of three parallel runs. The bars represent the standard deviation. The temperature downshift turns out to have a statistically significant effect (*p* < 0.05). Different lower letters indicate significant differences (Tukey’s test *p* < 0.05) between cold stress treatments and the control variants at the same period of cultivation.

The final stage of the TCA cycle includes the reversible oxidation of malate to oxaloacetate, catalyzed by MDH, which uses the reduction in NAD^+^ to NADP [47]. In all variants, a significantly lower level of activity was observed in old cells than in young cells (Figure 7).

In conditions of stress (15 and 6 °C), the activity of the enzyme demonstrated a marked temperature dependence. A higher level of the enzyme was observed at a stress of 6 °C for all variants of the model strains from the exponential and stationary phases (Figure 7). The temperature characteristic of the strain did not affect the changes in MDH. In the exponential phase, an increase of approximately 1.1- and 1.2-fold in MDH activity was observed in both the psychrotolerant and the two mesophilic strains (Figure 7A–C). Furthermore, exposure to stress during the stationary phase results in a significantly weaker response compared to that observed in young cells (Figure 7D–F).

Cold stress was found to significantly increase MDH activity by 25–35% in cultures exposed to 6 °C, compared to the control group (Supplementary Figure S6). At 15 °C, the response was less pronounced, with activity levels showing an increase of approximately 10–20% compared to untreated cultures. Similar patterns were observed during both growth phases.

## 3. Discussion

Temperature is an important factor in the growth and development of ectothermic organisms, such as fungi. In conditions of low temperatures or temperature fluctuations, they must maintain the structure of their cellular macromolecules while also sustaining a balance between energy generation and utilization processes. There is a growing consensus that cold treatment induces oxidative stress in all aerobic organisms, causing significant damage to proteins, lipids, and nucleic acids. These events can also contribute to ageing processes [1,5,16,43,49,50]. Although several studies have described the physiological and morphological adaptations that enable fungi to survive in a wide temperature range, little is known about how their metabolism responds to sudden temperature changes. Monitoring the metabolic activity of organisms exposed to low temperatures reveals that the activity of enzymes involved in catabolic processes can increase or change, leading to alternations in metabolic pathway patterns [51].

Our previous research demonstrated that long- and short-term temperature downshifts resulted in noticeable changes in stress biomarkers and antioxidant defences in the three penicillium strains isolated from Antarctic and temperate habitats [42,43,44]. Furthermore, the reported data provided evidence of accumulating oxidative damage during growth phases. The present study builds on this research by examining the metabolic response to cold stress in the same three penicillium strains belonging to different thermal classes (one psychrotolerant and two mesophilic). Overall, the results revealed dependence on stress level, growth phase, and temperature preference of the strain.

### 3.1. Cold Stress Affected the Enzyme Activity of Carbohydrate Metabolism

The first finding of the present study indicates an increase in the activity of the tested enzymes in both the glycolysis and TCA cycle pathways when they are subjected to cold-induced oxidative stress. A temperature decrease to 6 °C revealed more pronounced changes than a decrease to 15 °C. It should be noted that the observed results were accompanied by a similar trend in the activities of superoxide dismutase (SOD) and catalase (CAT) evaluated in these strains under cold stress [42,43].

#### 3.1.1. Changes in the Enzyme Activity of Glycolysis

Glycolysis is the central pathway of carbohydrate metabolism and occurs in the cytosol of all cells. This metabolic pathway comprises a series of biochemical processes that support the growth, reproduction and environmental interactions of fungi. HK plays a vital role in controlling energy metabolism by influencing the rate of glycolysis [46]. The formation of pyruvate, which is essential for the TCA cycle, depends on enzyme levels. The increased activity of the enzyme under cold shock conditions (15 °C and 6 °C) in the strains studied by us is likely due to the greater energy requirements in such extreme conditions. Enhanced HK activity is probably part of the fungal cell defence mechanism against cold stress. Evidence suggests that downregulation of HK accelerates ROS generation and increases stress levels in vivo and in vitro in higher eukaryotic cells [52,53] that significantly affects growth phase duration and reduces lifespan [54].

Furthermore, there are reports suggesting a correlation between accelerated trehalose synthesis under stressful conditions and the expression of HK in fungi and plants. Our previous studies have shown that exposure to cold stress increases the levels of reserve carbohydrates (such as trehalose and glycogen) within cells during exponential and stationary growth phases [42,43]. The relationship between HK enzyme activity and trehalose synthesis in yeast cells has also been proven. Yan et al. [55] found that adding trehalose to the cultivation of *Pleurotus ostreatus* under temperature stress altered the glycolytic enzyme profile. In this situation, an increase in the transcription level of the HK gene has been observed, while the transcription level of the phosphofructokinase has decreased.

Data related to filamentous fungi on changes in the activity of the enzyme HK under stress conditions are very scarce. More research on this topic has been published on plants. For example, HK1 acts as a key positive regulator of the cellular response to abiotic stress in *Arabidopsis thaliana* [56] and *Fragaria vesca* [57]. It is assumed that the enzyme is involved in the defence response of plants subjected to cold stress.

G6PDH initiates the PPP, which is also a major component of cellular metabolism. The PPP plays a key role in maintaining the carbon balance, providing precursors for nucleotide and amino acid synthesis, and protecting cells from oxidative stress [46]. As part of a defensive response to cold stress, G6PDH synthesis and NADPH production are triggered, improving antioxidant capacity and helping to eliminate excessive ROS produced by NADPH oxidase, ultimately reducing oxidative damage [58].

The present study also demonstrated a positive correlation between cold exposure and G6PDH activity. The stress level-dependent enhancement of G6PDH corroborated our previous results concerning a rise in oxidative stress biomarkers [42,43]. Significant levels of the enzyme have been identified in response to different stress factors. As posited by Kelavkar and Chhatpar [59], elevated levels of G6PDH were observed in *Aspergillus repens* under conditions of salt stress. The presence of paraquat and H_2_O_2_ has been demonstrated to trigger increased G6PDH activity in filamentous fungal cells [60]. The role of G6PDH as an antioxidant enzyme in fungi has been recognized in Pleurotus ostreatus [61] and *Fragaria ananassa* cv. Benihoppe [62]. The authors suggested that G6PDH overexpression reduces cold-induced ROS generation and membrane lipid peroxidation, contributing to cold tolerance. As demonstrated by Nóbrega-Pereira et al. [63], the enhanced level of G6PDH in mice led to the revelation of elevated levels of NADPH and augmented production of nucleotide precursors, which in turn protected the cells from the deleterious effects of ROS. Moreover, a manifold increase in the activity of PPP has been established. To ensure the metabolic reroute from glycolysis to PPP, the cells cooperate with different regulatory mechanisms, including post-translational modifications of G6PDH activity [64]. This mechanism averts G6PDH from becoming the rate-limiting enzyme in the PPP. Analogous results have been reported in *Xenopus laevis* cells [65], mammals [66], cancer cells [67], and various other entities.

The results of the present study revealed that the metabolic response of penicillium strains to cold stress is also associated with the GAPDH enzyme. This enzyme facilitates the reversible conversion of glyceraldehyde 3-phosphate into 1,3-bisphosphoglycerate [46]. Moreover, recent investigations reveal that GAPDH is implicated not only in glycolysis, but also plays an important role in response to abiotic stress [68,69]. However, data on the effects of low temperatures on GAPDH production by other filamentous fungi are extremely limited. Treating the fungus *Lentinus polychrous* with low and high temperature stress caused a six-fold increase in GAPDH enzyme activity [70]. Enhanced enzyme activity has also been observed under other types of stress. GAPDH constitutes a tolerance mechanism in *Aspergillus nidulans* to H_2_O_2_ stress [71] and in *Schizosaccharomyces pombe* against peroxide stress [72].

Analyzing the enhanced activity of glycolytic enzymes, HK, G6PDH, and GAPDH in the three penicillium strains subsequent to a temperature downshift, we found that this cellular response was consistent with our previous research on fungal cell responses, which demonstrated an increase in the synthesis of storage carbohydrates (trehalose and glycogen) and upregulation of gluconeogenesis [42,43]. Accumulation of reserve carbohydrates has been demonstrated to alleviate oxidative stress by suppressing the increases in glycolysis and thus enhancing PPP activity and NADPH production [55,64]. Suppression of glycolysis and the redirection of metabolite flow to the PPP can occur within seconds, providing rapid cellular protection and enabling high metabolic activity to be maintained [64].

#### 3.1.2. Changes in Enzyme Activity of the TCA Cycle

The current work established how the three rate-limiting enzymes of the TCA cycle (ICDH, SDH and MDH) are affected by low-temperature treatment. According to our findings, the TCA cycle plays an important role in defence against cold stress. Noster et al. [73] reported that oxidative stress increases the TCA cycle activity and the level of these enzymes.

ICDH is a metabolic enzyme that catalyzes the oxidative decarboxylation of isocitrate to α-ketoglutarate, transforming oxidized NAD (NADP) into NADH (NADPH) in the process [47]. This enzyme is recognized as a key rate-limiting factor in the TCA cycle [74]. Furthermore, it controls the carbon flow between theTCA cycle and the switch to the glyoxalate cycle. As a source of NADPH, the enzyme also plays a protective role against ROS in cells. ICDH plays an antioxidant role in *Salmonella enterica* serovar Typhimurium by accumulating NADPH during oxidative stress [73]. A similar effect of this enzyme has been evaluated in response of *Zea mays* in response to drought stress [74]. The role of ICDH in the strategy against oxidative stress has been demonstrated in a range of organisms, including *Pseudomonas fluorescens* [75], Drosophila [76], rat liver cells [77], and cancer cells [78]. Overexpression of ICDH caused a significant increase in oxidative stress tolerance in *Caenorhabditis elegans* [79].

Next, the results obtained in this study demonstrate that an increase in SDH synthesis takes place in the fungal cultures of the tested penicillium strains when they are subjected to cold stress. This enzyme participates in the growth rate control of pathogenic fungi under various stress conditions. A lower SDH level has been linked to a faster generation of ROS and increased sensitivity to oxidative stress in the cells of *Beauveria bassiana* [80,81] and *Botrytis cinerea* [82]. There is no information in scientific literature on the behaviour of SDH in filamentous fungi during treatment with low temperatures. Therefore, a comparison can be made with studies conducted on higher eukaryotes. Correlation between SDH activity and higher tolerance to cold stress has been reported in a range of plant species [83].

Following the SDH reaction in a cold stress response, the alterations in MDH were presented (Figure 7). The strains used in the present study demonstrated increased enzyme activity during the temperature downshift period compared to the control variant, depending on the degree of stress. This escalation coincided with the elevated oxidative stress biomarkers previously evidenced in the test fungal cultures under low temperature treatment [42,43]. The data obtained in this study can be compared with the results reported in the existing literature on plant cells. Several studies have demonstrated an increase in MDH in response to biotic and abiotic stresses [84,85]. The positive role of MDH in defence against accelerated ROS generation has been detected in *Arabidopsis thaliana* [86]. The addition of malate to plant cells has been shown to increase the level of ROS and to cause programmed cell death. Moreover, TCA cycle regulation in the marine bacterium *Loktanella salsilacus* under cold stress is due to the synthesis of additional TCA enzyme molecules rather than changes in enzyme activity [87].

Taken together, the data presented in the current study provide information on the complicated relationship between the TCA cycle and ROS homeostasis in filamentous fungi. Consequently, the TCA cycle serves as an important component in the maintenance of cellular redox balance through its ability to produce and neutralize ROS [88]. The upregulation of the main degrading enzymes in the TCA cycle leads to a reduction in the rate of ROS generation and modulates the cell resistance to oxidative stress [73].

It is noteworthy that the tendency toward higher enzyme activity compared to the control variant persisted even after the temperature was returned to the optimal level. In fact, at this stage, the difference in activities between the treatment group and the control group decreases. The reverse effect is noticeable, particularly after four hours have passed since the stress stopped. Similar behaviour was observed in our previous study concerning changes in biomarkers of oxidative stress and antioxidant defence enzymes [42,43]. Additionally, stopping cold exposure and returning to an optimal temperature did not reveal any differences related to the growth phase. It is likely that the ongoing accumulation of trehalose and glycogen, along with elevated levels of superoxide dismutase (SOD) and catalase (CAT), helped maintain the metabolic activity of the treated fungal cultures for a certain period. Similar irreversible damage has been evaluated in *Flammulina velutipes* and *Mrakia psychrophila* after low temperature exposure [41,89]. In response to cold stress, they modified the molecular content in their complex protein networks.

### 3.2. Cold Stress Response and Temperature Preference of the Tested Strains

Secondly, we presented data concerning the correlation between the cold stress response of the penicillium strains and their temperature characteristics, as well as the significance of the habitat from which they were isolated. Strain-dependent variations in the response to cold stress have been demonstrated regarding enzymes of carbohydrate metabolism. Elevated enzyme activity was more closely related to the temperature preference of the strains than to the site of their isolation. The psychrotolerant strain *P. olsonii* p14 demonstrated the best adaptation to low temperatures compared to the mesophilic fungi. This strain exhibited the most significant increase in glycolytic pathway enzymes, particularly when treated at 6 °C. Additionally, the established rerouting to the PPP was more pronounced in *P. olsonii* p14 than in the two mesophilic strains. The noted trends were consistent throughout both phases of development. The present findings are consistent with the behaviour of the studied strains under low-temperature stress conditions, as demonstrated in our previous studies [42,43]. When the temperature was reduced from the optimal level to 15 or 6 °C, *P. olsonii* p14 exhibited a smaller decline in biomass, a smaller increase in damaged proteins and a greater capacity to accumulate reserve carbohydrates than the mesophilic strains (Antarctic and temperate). Furthermore, exposure to low-temperature stress resulted in a smaller increase in antioxidant enzyme activity in the psychrotolerant strain than in the mesophilic strains.

The role of glycolysis in the cellular response to low-temperature stress in fungi remains completely unclear. Even less is known about the significance of the strain’s temperature characteristics when it is exposed to cold stress. Similarly to our findings, Abu Bakar et al. [90] demonstrated that there is no correlation between the geographic origin of fungal strains and the response to cold stress in members of the genus *Pseudogymnoascus*. The response to this treatment is dependent upon the temperature characteristics of the strain. The Antarctic psychrophilic isolate *Pseudogymnoascus* sp. sp3 showed a different stress response profile compared to mesophilic strains. Furthermore, an activation of the pentose phosphate pathway (PPP) and NADPH synthesis has been demonstrated in the psychrophilic strain *Saccharomyces cerevisiae* in conditions of oxidative stress [91]. Similar data have been reported for Rodoturula frigidialcoholis [92] and Mucor lusitanicus [93].

In support of this suggestion, some studies about psychrophilic bacteria have been published. For example, *Shewanella psychrophile* has been observed to modify its metabolic efficiency at suboptimal and supraoptimal temperatures [94]. In the study by García-Descalzo et al. [95], the behaviour of psychrotolerant and mesophilic strains of the genus *Shewanella* was examined under conditions of cold stress. Analysis has revealed a more flexible adaptive response in psychrotolerant *S. frigidimarina* to a sharp decrease in temperature in comparison to its mesophilic counterpart.

In contrast, the present study demonstrated that the changes in the activity of enzymes of the TCA cycle in response to cold stress were independent of temperature characteristics or geographic origin. The observed increase in ICDH, SDH, and MDH activity was revealed to be completely determined by the level of stress present. It is worth noting that there is a lack of published data reporting differences in the cell responses of psychrophilic and mesophilic microorganisms (including fungi) to cold stress concerning TCA cycle enzymes. According to Tsuji [38], cold stress induces the accumulation of high levels of TCA cycle metabolites in two *Mrakia blollopis* strains: one capable of growing at sub-zero temperatures and the other not. However, both strains show marked differences in their metabolite responses, particularly in the accumulation of lactic acid, aromatic amino acids, and polyamines. The absence of strain-dependent variation in TCA-cycle enzyme activities suggests the presence of more complex relationships between the strain origin and response to cold stress.

Our results confirm that the cellular response to cold stress involves metabolic changes. In their evolution, cold-adapted fungi have developed mechanisms for survival in conditions of a sharp decrease in growth temperature. Physiological adaptation includes the accumulation of trehalose, glycogen, polyols, antifreeze proteins, and extracellular exopolysaccharides [96]. A significant change in the production of unsaturated fatty acids and a change in mRNA structure have been demonstrated [89]. Abu Bakar [89] showed that *Pseudogymnoascus* spp. respond to cold stress by consuming nutrients available to promote cell damage and repair while requiring minimal cell growth. They established a modulation in carbon and amino acid metabolism to minimize energy use for culture development. The Antarctic *sp3* isolate showed upregulation of glycolysis/gluconeogenesis pathways. Additionally, gene expression changes, production of cold-adapted and cold-active enzymes were definite as a part of molecular adaptation [97]. Cold-active enzymes possess a number of properties that facilitate enhanced catalytic efficiency and substrate utilization under low temperatures. In the review of Yusof et al. [97], the synthesis of cold-active enzymes (chitinase, esterase, exo-β-1,3-glucanase, α-amylase, etc.) by cold-adapted fungi has been reported.

### 3.3. Growth Phase-Dependent Cold Stress Response

#### 3.3.1. Growth Phase-Dependent Changes Under Optimal Conditions

The main finding of the present study is that the metabolite response of cells to cold stress depends on the growth phase of the model penicillium strains. Under optimal cultivation conditions, enzyme activity in the glycolytic pathway and the TCA cycle was significantly lower in old cells than in young cells of the studied strains The transition to gluconeogenesis in the stationary phase, which is associated with the accumulation of reserve carbohydrates, is likely the reason for this drastic difference in activity [55]. The inverse relationship between the activity of the enzyme HK and the synthesis of trehalose in yeast cells has been proven by Peixoto and Panek [98]. The authors found reduced enzyme activity in *Saccharomyces cerevisiae* during the stationary phase, when the accumulation of reserve carbohydrate begins. The maximal activities of glycolytic enzymes in Ustilago maydis also demonstrated growth phase-dependent modification [99]. There is evidence that the enzyme HK is associated with cellular ageing processes. This enzyme has been shown to play a role in controlling apoptosis in *Aspergillus nidulans* cells [100]. A growth phase-dependent decrease in the activity of key metabolic enzymes has been reported in *Saccharomyces cerevisiae* [101] and *Aspergillus nidulans* [102] under optimal culture conditions. Additionally, a considerable decrease in the activity of TCA cycle dehydrogenases has been established in aged *Saccharomyces cerevisiae* cells [103]. According to den Ridder et al. [101], genetic reduction in glycolysis during the stationary phase takes place at the post-translational level rather than at the transcriptional level.

#### 3.3.2. Growth Phase-Dependent Changes Under Cold Stress Conditions

Despite the observed large differences in enzyme activity between younger and older cells, the current study’s results indicated that cold stress enhances enzyme activity in both growth phases. The cell response included the induction of enzyme activities regardless of the age of the cultures. However, the increases in exponential growth phase cultures were more significant and sustained than those in stationary phase cultures. Activation of key metabolic pathways likely increases the defence system and the ability to survive stress even in older cells. Similar studies on microorganisms, including fungi, are extremely rare. The role of glycolytic enzymes in responding to oxidative stress and modulating cellular lifespan has been studied in higher eukaryotic cells. The data obtained indicate that the modulation of glycolytic pathways plays an active role in regulating oxidative stress and ageing in animals. Evidence suggests that enhanced glycolysis could affect cellular lifespan by reducing oxidative stress in cancer cells [104]. In this regard, information exists on the function of NAD^+^-dependent glycolytic enzymes in response to stress and ageing in human cells [105], as well as in yeast, worms, flies and mice [106].

Under oxidative stress conditions, enzymes of the TCA cycle play a key role in regulating cellular ageing rates. For instance, SDH is particularly crucial in this regard [107]. Disruption of aconitase expression and inhibition of SDH due to oxidative damage have been found to cause significant alterations in cell metabolism, accelerating the ageing process. Additionally, ICDH in *Caenorhabditis elegans* is involved in controlling the ageing process and mitigating oxidative stress [79]. Mitochondrial TCA cycle metabolites such as citrate and fumaric acid have been shown to slow down cellular ageing in Drosophila [108]. Elevated levels of α-ketoglutarate have also been linked to delayed age-related phenotypic changes in *C. elegans*, Drosophila, and other organisms [76]. The authors emphasize the similarity of the mechanisms controlling lifespace in flies, invertebrates, and vertebrates.

## 4. Materials and Methods

### 4.1. Fungal Strains and Cultivation

The fungal strains, *Penicillium olsonii* p14 and *Penicillium waksmanii* m12, isolated from Antarctic soils, as well as strain *Penicillium rugulosum* t35, isolated from temperate Bulgarian soil samples (the region Knyajevo, Sofia), were used in the experiments. *P. olsonii* p14 is a psychrotolerant strain, with an optimal growth temperature of 20 °C; *P. waksmanii* m12 and *P. rugulosum* t35 are mesophilic strains with an optimal temperature of 30 °C [109]. All strains belong to the Mycological collection at the Stephan Angeloff Institute of Microbiology, Sofia. Long-term preservation was carried out in the Microbank system (Prolab Diagnostics, Richmond Hill, ON, Canada) containing sterile vials that contain 25 porous, coloured beads and a cryopreservative fluid at −80 °C. Before use, the conidiospores were grown on Beer agar at 28 °C for 7 days.

The composition of the culture medium AN-3 was described in Gocheva et al. [42]. The cultivation in 3 L bioreactors (ABR-09, equipped with temperature, pH, and automatic dissolved oxygen monitoring equipment and a control system) was used as previously described [42]. The submerged cultivation was carried out in two stages. The first stage was performed in 500 mL Erlenmeyer flasks (74 mL medium AN-3 and 6 mL spore suspension at a concentration of 2 × 10^8^ spores/mL) at the optimal temperature (20 °C for *P. olsonii* p14 and 30 °C for *P. waksmanii* m12 and *P. rugulosum* t35, respectively), for 48 h on a rotary shaker (220 rpm).

For bioreactor cultures, 200 mL of the seed culture was brought into the 3 L bioreactor, containing 1800 mL of the medium AN-3. The cultures were grown at the corresponding optimal temperatures with a stirrer speed of 400 rpm, an air flow of 0.5 vvm, until they reached the middle-exponential or middle-stationary growth phases. The dry weight thresholds for the exponential-phase cultures were 0.260, 0.340, and 0.460 g/100 mL for *P. olsonii* p14, *P. waksmanii* m12, and *P. rugulosum* t35, respectively. For the stationary-phase cultures, the thresholds were 1.562, 1.517, and 1.634 g/100 mL, respectively. Then, the temperature in the bioreactors was reduced to 15 °C or 6 °C for 6 h, followed by restoration of the optimal temperature for a further 4 h. The control variants were grown at the optimal temperature throughout the whole period. Enzyme activity was measured at every 2 h. The stages of the experimental design were presented in Figure 1.

### 4.2. Preparation of Cell Fractions

The cell fractions were prepared as previously described [110]. All steps were performed at 0–4 °C. In brief, the washed mycelium biomass was suspended in an isolation buffer containing 0.25 M sucrose, 5 mM EDTA, and 0.15% bovine serum albumin at pH 7.5. Then, the biomass was disrupted using an ULTRA-Turrax T25 IKA homogenizer (Staufen, Germany). The cell-free extract was then centrifuged at 15,000× *g* for 20 min at 4 °C. The resulting liquid fraction was used as the cell-free extract (CFE). Cytosol (Cyt) was obtained by centrifuging the 15,000× *g* supernatant at 144,000× *g* for 60 min. The 15,000× *g* precipitate was resuspended in the same volume of isolation buffer and centrifuged for 40 min at 30,590× *g*; then, the supernatant was discarded, and the crude mitochondrial pellets (CMP) were carefully washed with SEM buffer (0.25 M sucrose, 5 mM EDTA, and 10 mM 3-(N-morpholino)propanesulfonic acid [MOPS]/KOH], pH 7.5). This procedure was repeated three times. The collected mitochondria were then resuspended in deionized water and lysed by freezing and thawing. After treating the intact mitochondria and centrifuging them at 144,000× *g* for 1 h, the mitochondrial fraction was obtained.

### 4.3. Enzyme Activity Determination

HK (EC 2.7.1.1.), G6PDH (EC 1.1.1.49.), GAPDH (EC 1.2.1.12) of the cytosolic fraction and ICDH (EC 1.1.1.41.), SDH (EC 1.3.99.1.), and MDH (EC 1.1.1.37.) from mitochondrial fraction were measured spectrophotometrically using Shimadzu UV-Vis Spectrophotometer (A116350) equipped with thermostated cell holder.

Protein was estimated by the Lowry procedure [111] using crystalline bovine albumin as standard.

#### 4.3.1. Enzyme Activity of Glycolysis

HK, G6PDH, and GAPDH were determined by the methods of Bergmeyer [112]. HK activity was measured through NADP^+^ reduction in a glucose-6-phosphate dehydrogenase-coupled reaction at 30 °C. The reaction mixture (1 mL quartz cuvette) contained 10 mM glucose, 20 mM Tris/HCl buffer, pH 7.5, 7.5 mM MgCl_2_, 0.5 mM ATP, 0.65 mM NADP^+^, 2 units glucosed-phosphate dehydrogenase, and 50 μL of a suitably diluted cytosolic fraction. Absorbance was continuously recorded at 340 nm for 10–15 min.

G6PDH activity was measured by spectrophotometric quantitation of reduced NADP^+^ at 30 °C and λ = 340 nm. The reaction mixture contained 50 mM triethanolamine buffer pH 7.5, 10 mM MgCl_2_, 0.083 mM NADP and 0.833 mM glucose 6-phosphate. One G6PDH unit was defined as the amount of enzyme catalyzing the reduction of 1 µmol of NADP/min under the assay conditions.

GAPDH activity was measured (λ = 340 nm) in 3 mL reaction mix comtained 83 mM triethanolamine, 6.7 mM 3-phisphoglyceric acid, 3 mM L-cysteine, 2 mM Mg_2_SO_4_, 0.1 mM NADH, 1.1 mM ATP, 5 U 3-phosphoglyceric phosphophructikinase, and 0.05 mL cytosol fraction. One GAPDH unit was defined as the amount of enzyme catalyzing the reduction of 1 µmol of 3-phosphoglycerate to D-glyceraldehyde 3-phosphate per min at pH 7.6 at 30 °C.

#### 4.3.2. Enzyme Activity of TCA Cycle

For estimation of ICDH activity, the methodology of Kornberg [113] was followed. The reaction mixture contained 40 mM Tris-HCl (pHI 7.6), 4 mM MgCl_2_, 2.5 mM trisodium DL-isocitrate, 0.25 mM NAD+, 1 mM KCN, and 0.1 mL enzyme. The assay was performed at 25 °C at 340 nm between 15 sec intervals.

SDH was estimated following Veeger et al. [114]. Assay mixture contained 0.2 M Na-phosphate buffer pH 7.6, 30 mM EDTA, 75 mM potassium ferricyanide, 3% serum albumin, 0.4 M succinate, and 0.1 mL mitochondrial fraction. Absorbance was recorded at 30 °C at 340 nm for 3 min with 30 s intervals.

MDH in the mitochondrial fraction was assay by method of Kornberg and Horecker [115]. The reaction medium contained 100 mM glycine–NaOH buffer (pH 9.5), 0.4 mM NAD^+^, 3.0 mM L-malate, and 0.1 mL enzyme. The readings were taken at 340 nm at 30 °C over a period of three minutes, with 30 s intervals.

### 4.4. Statistical Evaluation of the Results

The results obtained in this investigation were evaluated through at least three repeated experiments using three parallel runs and the reported values to represent the mean. The error bars indicate the standard deviation (SD) of the mean of triplicate experiments. The data were analyzed using One-Way analysis of variance (ANOVA), followed by Tukey’s test. For the statistical processing of the data, the version of the ANOVA software built into the Origin programme (OriginPro 2019b, 64-bit) was used.

## 5. Conclusions

The present study provides new information about the metabolic response of filamentous fungi isolated from extremely cold environments to low-temperature stress and its relationship to ageing. The results corroborate the findings of our earlier studies, which demonstrated that low temperatures trigger oxidative stress in *Penicillium* strains originating from permanently cold and temperate climates (Antarctica and Bulgaria, respectively) [57,65]. Our present data demonstrated that the transient temperature downshift activated the glycolysis and TCA cycle in a temperature-dependent manner. Furthermore, a shift in metabolism from glycolysis to the PPP was observed under stress conditions. The established enhancement in enzyme activity of glycolysis was affected by the temperature characteristic of the strain, rather than the geographic region of isolation. Among the strains studied, the psychrotolerant *P. olsonii* p14 displayed the highest level of adaptation to low temperatures relative to the two mesophilic fungi. In contrast, the activity of TCA cycle enzymes in response to cold stress remained unaffected by temperature requirements or geographic origin. The key result of this study reveals that cold stress enhances enzyme activity in both growth phases. The stimulation of glycolysis and the TCA cycle takes place regardless of the age of the cultures. Exponential growth phase cultures demonstrated a more substantial and prolonged response to cold stress compared to stationary phase cultures. Taken together, the present findings and our previous data provide insights into the role of cold-induced oxidative stress in disrupting fungal cell homeostasis and its relationship with the growth phases. This information could significantly contribute to the prediction and management of fungal pathogen behaviour, with applications for both plant and human fungal infections.

## Data Availability

Data are contained within the article.

## Supplementary Materials

The following supporting information can be downloaded at https://www.mdpi.com/article/10.3390/ijms26199308/s1.

## Abbreviations

The following abbreviations are used in this manuscript:

Younger cells	cells from exponential-phase cultures
Older cells	cells from stationary-phase cultures
ATP	adenosine triphosphate
NADPH	nicotinamide adenine dinucleotide phosphate (reduced form)
ROS	reactive oxygen radicals
^●^O_2_^−^	superoxide radicals
H_2_O_2_	hydrogen peroxide
OH•	hydroxyl radicals
^1^O_2_	singlet oxygen
HK	hexokinase
G6PDH	glucose-6-phosphate-dehydrogenase
GAPDH	glyceraldehyde-3-phosphate dehydrogenase
ICDH	isocitrate dehydrogenase
SDH	succinate dehydrogenase
MDH	malate dehydrogenase
TCA cycle	tricarboxylic acid cycle
