# Supplementary material for: Growth Phase-Dependent Changes in the Carbohydrate Metabolism of Penicillium Strains from Diverse Temperature Classes in Response to Cold Stress
Source: Int J Mol Sci. 2025 Sep 24;26(19):9308. doi: 10.3390/ijms26199308 (PMC12525117; doi:10.3390/ijms26199308)
Supplement: Supplementary file 1 [file ijms-26-09308-s001.zip › ijms-3821793-supplementary.pdf]

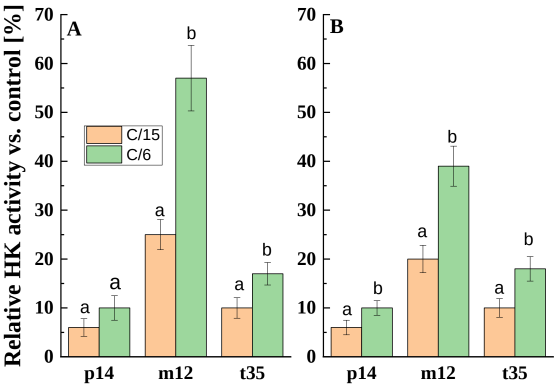

Figure S1. Percentage increase in hexokinase activity in cultures subjected to cold stress at 6 or 15°C, relative to the control (at the respective optimal temperatures), over a duration of 6 hours. A – cultures from the exponential growth phase; B – cultures from the stationary growth phase. The relative activity was calculated from the data, presented in Figure 2. Different lower letters indicate significant differences (Tukey's test $p<0.05$) between cold stress treatments and the control variants at the same period of cultivatio*n*.

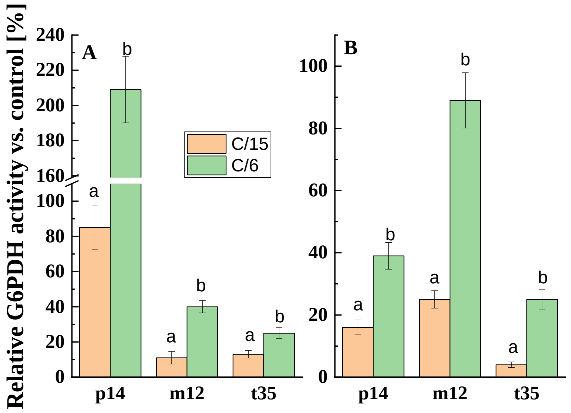

Figure S2. Percentage increase in glucose-6-phosphate dehydrogenase activity in cultures subjected to cold stress at 6 or 15°C, relative to the control (at the respective optimal temperatures), over a duration of 6 hours. A – cultures from the exponential growth phase; B - cultures from the stationary growth phase. The relative activity was calculated from the data, presented in Figure 3. Different lower letters indicate significant differences (Tukey's test $p<0.05$) between cold stress treatments and the control variants at the same period of cultivatio*n*

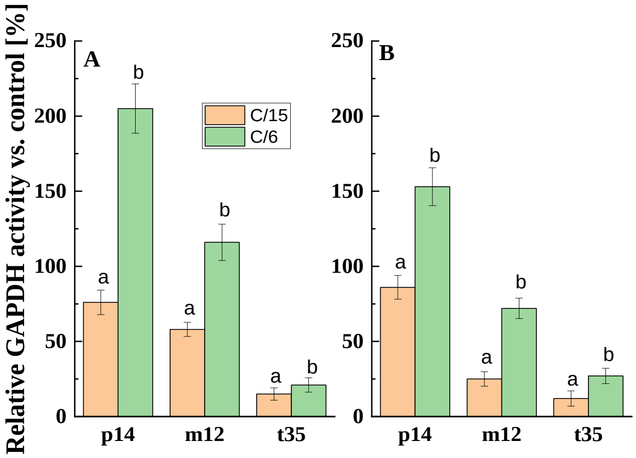

Figure S3. Percentage increase in glyceraldehyde phosphate dehydrogenase activity in cultures subjected to cold stress at 6 or 15°C, relative to the control (at the respective optimal temperatures), over a duration of 6 hours. A – cultures from the exponential growth phase; B - cultures from the stationary growth phase. The relative activity was calculated from the data, presented in Figure 4. Different lower letters indicate significant differences (Tukey's test p<0.05) between cold stress treatments and the control variants at the same period of cultivation

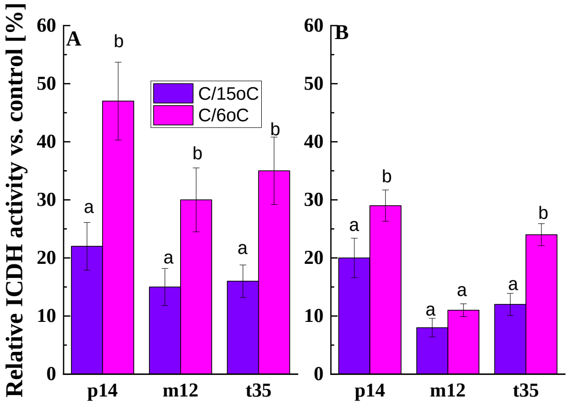

Figure S4. Percentage increase in isocytrate dehydrogenase activity in cultures subjected to cold stress at 6 or 15°C, relative to the control (at the respective optimal temperatures), over a duration of 6 hours. A – cultures from the exponential growth phase; B - cultures from the stationary growth phase. The relative activity was calculated from the data, presented in Figure 5. Different lower letters indicate significant differences (Tukey's test p<0.05) between cold stress treatments and the control variants at the same period of cultivation

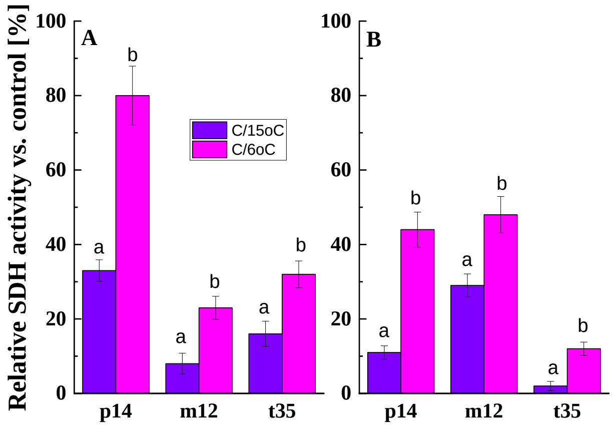

Figure S5. Percentage increase in succinate dehydrogenase activity in cultures subjected to cold stress at 6 or 15°C, relative to the control (at the respective optimal temperatures), over a duration of 6 hours. A – cultures from the exponential growth phase; B - cultures from the stationary growth phase. The relative activity was calculated from the data, presented in Figure 6. Different lower letters indicate significant differences (Tukey's test $p<0.05$) between cold stress treatments and the control variants at the same period of cultivatio*n*

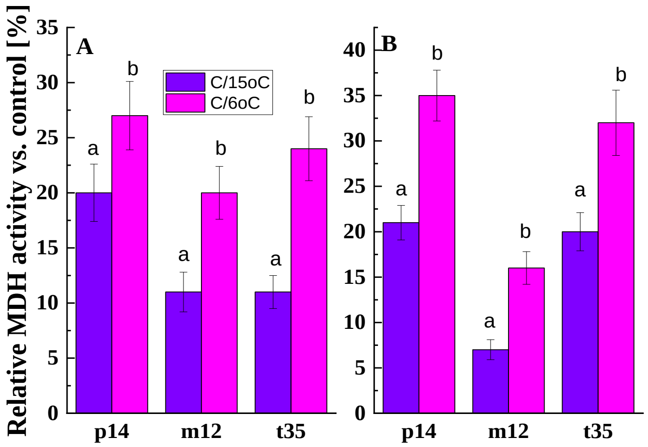

Figure S6. Percentage increase in malate dehydrogenase activity in cultures subjected to cold stress at 6 or 15°C, relative to the control (at the respective optimal temperatures), over a duration of 6 hours. A – cultures from the exponential growth phase; B - cultures from the stationary growth phase. The relative activity was calculated from the data, presented in Figure 7. Different lower letters indicate significant differences (Tukey's test $p<0.05$) between cold stress treatments and the control variants at the same period of cultivatio*n*
